# Evaluation of Gait Phase Detection Delay Compensation Strategies to Control a Gyroscope-Controlled Functional Electrical Stimulation System During Walking

**DOI:** 10.3390/s19112471

**Published:** 2019-05-30

**Authors:** Nicole Zahradka, Ahad Behboodi, Henry Wright, Barry Bodt, Samuel Lee

**Affiliations:** 1 Biomechanics and Movement Science Program, University of Delaware, Newark, DE 19713, USA; nzahrad1@jhu.edu (N.Z.); ahadbeh@udel.edu (A.B.); 2 Department of Physical Therapy, University of Delaware, Newark, DE 19713, USA; henryw@udel.edu; 3 Biostatistics Core Facility, College of Health Sciences, University of Delaware, Newark, DE 19713, USA; babodt@udel.edu; 4 Shriners Hospitals for Children, Philadelphia, PA 19140, USA

**Keywords:** functional electrical stimulation (FES), gait phase detection (GPD), finite-state control

## Abstract

Functional electrical stimulation systems are used as neuroprosthetic devices in rehabilitative interventions such as gait training. Stimulator triggers, implemented to control stimulation delivery, range from open- to closed-loop controllers. Finite-state controllers trigger stimulators when specific conditions are met and utilize preset sequences of stimulation. Wearable sensors provide the necessary input to differentiate gait phases during walking and trigger stimulation. However, gait phase detection is associated with inherent system delays. In this study, five stimulator triggers designed to compensate for gait phase detection delays were tested to determine which trigger most accurately delivered stimulation at the desired times of the gait cycle. Motion capture data were collected on seven typically-developing children while walking on an instrumented treadmill. Participants wore one inertial measurement unit on each ankle and gyroscope data were streamed into the gait phase detection algorithm. Five triggers, based on gait phase detection, were used to simulate stimulation to five muscle groups, bilaterally. For each condition, stimulation signals were collected in the motion capture software via analog channels and compared to the desired timing determined by kinematic and kinetic data. Results illustrate that gait phase detection is a viable finite-state control, and appropriate system delay compensations, on average, reduce stimulation delivery delays by 6.7% of the gait cycle.

## 1. Introduction

Functional electrical stimulation (FES) is the application of electrical stimulation to assist functional movements such as standing [1,2,3], walking [4,5,6], and grasping [7,8] in individuals with upper motor neuron lesions. FES systems are used as neuroprosthetic devices [9] in the rehabilitative settings and the delivery of stimulation relies on open-or closed-loop controllers. While adaptive closed-loop control is the ideal controller for FES systems, because it provides modulation of stimulation parameters to achieve desired movements, few stable closed-loop feedback systems have been developed [10,11,12,13]. Open-loop controlled FES systems are typically used in clinical settings because they are easy to don/doff; however, provide less accurate movement control because these systems rely on manual input to trigger the delivery of stimulation [14]. The Parastep I (Sigmedics, Inc., Fairborn, OH, USA) and RehaStim (Hasomed Inc., Germany) are two examples of commercially-available FES systems that utilize open-loop controllers. Both systems require pushing a button to start the stimulation program and, once initiated, stimulation parameters such as timing are fixed and require the user to try to match their gait speed to the timing of the stimulation program.

Finite-state-controlled FES systems provide an intermediate solution in stimulation delivery by incorporating a level of accuracy exhibited in closed-loop controllers, while utilizing a minimized number of sensors to maintain the clinically-friendly setup associated with open-loop control. This type of controller, technically a closed-looped control because of the feedback component, uses preset sequences of stimulation [15] that are triggered when specific conditions are met and require minimal sensors to collect feedback. 

FES systems have been utilized in walking interventions to restore movement during gait [16,17,18,19,20,21,22]. To elicit restorative walking patterns, several FES systems implement finite-state controllers to trigger stimulation and utilize external sensors to provide feedback on different parameters during the gait cycle [23]. Technologies such as force sensing resistors, accelerometers, and micro-electromechanical system devices have been utilized in research-based and commercial devices to detect gait events [24], measure activity [25], quantify spatiotemporal variables of gait [26], and to track and analyze gait kinematics [27]. Some commercially-available FES systems that take advantage of such technologies are the Odstock Dropped Foot Stimulator systems (Biomedical Engineering and Medical Physics, Salisbury, UK), Respond II Select (Medtronic Inc., Minneapolis, MN, USA), and Ness L300^®^ Plus (Bioness Inc, Valencia, CA, USA). These stimulators use sensors to detect distinct events during the gait cycle to control stimulation delivery. However, existing gait event-controlled FES systems are typically limited in the timing of the stimulation delivery because few [23,27] are capable of detecting all seven phases of gait [28]. For example, FES systems with the most basic gait detection methods, distinguishing only between stance and swing periods [29], cannot accurately target three muscle groups that typically begin to fire during mid-swing (hamstrings) and terminal swing (quadriceps femoris and gluteal muscles) [30]. With the timing of stimulation delivery playing a crucial role in FES interventions [31,32,33], the capability of detecting all seven phases of gait is needed to provide the amount of control necessary to deliver stimulation in a manner that is representative of typical muscle firing patterns.

In addition to the need for increased system control, delay compensations are necessary as FES system input delays may result in performance degradation [34] and potentially cause instability [35]. Previous work focused on compensating for electromechanical delay, the time lag between electrical activation of the muscle, and the onset of muscle force [36] to deliver stimulation at appropriate times with FES systems [37,38,39]. Sharma et al. developed a controller with a predictor term that actively compensated for electromechanical delay [34]. A PID controller with a delay compensation algorithm to adjust for electromechanical *and* communication delays controlled elbow position more accurately than the PID controller alone, especially when the combined delay exceeded 35 ms [40]. However, this composite delay compensation assumed communication delays to be fixed. FES system performance and timing accuracy of the stimulation signals prior to application is necessary to verify that changes observed during FES gait interventions are created as a result of the application of stimulation at the targeted times in the gait cycle.

Previous work has reported on gait phase detection (GPD) algorithm delays and alluded to the ability to implement gait phase detection as a trigger for accurately-timed delivery of stimulation [41,42,43,44,45]. Latencies are dependent on sensor/algorithm and have been reported with varied delays, such as having a delay range between 21.86 ms (midswing) to 64.46 ms (toe off) [42], delays less than 125 ms [41], and gait phase duration differences within 80 ms when compared to gait phase durations derived from the literatures [44]. Pappas et al. reported the reliability to detect four gait phases to be within 90 ms when their gait phase detection (GPD) system [24] was compared to phase detection by motion capture, while reporting the capacity of the combined GPD system and FES system to alter ankle trajectories [46]. Gait phase detection delays are the same order of magnitude as electromechanical delay (30–100 ms) [36]; there can be up to twice as much delay between the desired and realized action. Validation of gait phase detection delay compensation strategies to trigger FES systems and the timing accuracy of stimulation delivery has received less attention, even though optimization of stimulation delivery time is crucial in the success of FES interventions [31,32,33].

Our group has designed a real-time GPD system, with optimized sensor setup and data processing, that is capable of detecting seven phases of gait with 100% detection reliability [47]. Although minimal, gait phase detection onset differences were observed when compared to the timing of the motion capture derived gait phases. The purpose of this paper is to evaluate five finite-state FES system triggers, designed to compensate for the inherent gait phase detection delays, by comparing stimulation delivery time to the desired timing during walking in typically-developing (TD) children.

## 2. Materials and Methods

### 2.1. FES System

A multichannel surface FES system incorporated two inertial measurement units (IMUs) (Opal^TM^, APDM, Portland, OR, USA), two stimulators (RehaStim, Hasomed Inc., Magdeburg, Germany), and a custom gait phase detection (GPD) algorithm (LabVIEW, National Instruments, Austin, TX, USA) [47,48] capable of communicating with the stimulators. The IMUs were placed on the lateral shanks near the ankles; ipsilateral and contralateral shank angular velocity (gyroscope signal) was wirelessly streamed into the GPD algorithm, as the finite-state controller of the system. Predefined rules were established to distinguish between the 7 phases of gait: Loading Response (*LR*), Mid-Stance (*MSt*), Terminal Stance (*TSt*), Pre-Swing (*PSw*), Initial Swing (*ISw*), Mid-Swing (*MSw*), and Terminal Swing (*TSw*). The GPD system was validated during walking in typically-developing children; onset detection of gait phases determined by the GPD algorithm had root mean square errors that ranged from 35 ms (*MSw*) to 105 ms (*TSw*) when compared to motion capture [47].

Finite-state control of the FES system was achieved by using gait phased detection, determined by the GPD system, to trigger the stimulator. The software was programmed to trigger stimulation to five muscle groups based on typical muscle activity during self-selected walking (Table 1) [30].

**Figure 1:**
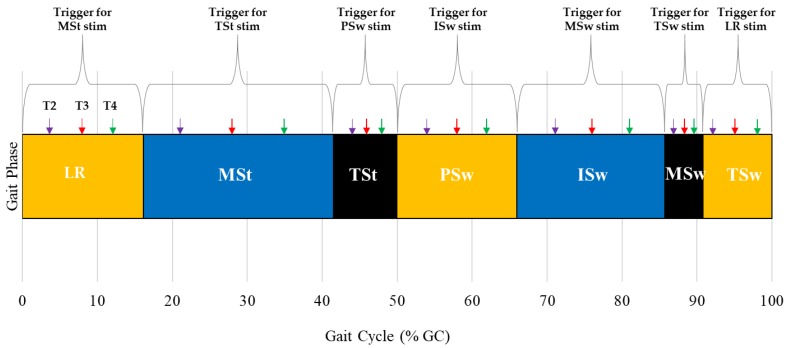
A representation of trigger timing for each gait phase. Purple, red, and green arrows illustrate the percentage delay added to gait phase onset for *T2* (25% gait phase duration), *T3* (50% gait phase duration), and *T4* (75% gait phase duration), respectively. *T2*–*T4* triggered stimulation (stim) for the upcoming phase. The same trigger condition was applied to all gait phases. *T2*, 25% gait phase duration delay trigger; *T3,* 50% gait phase duration delay trigger; *T4,* 75% gait phase duration delay trigger. *LR*—Loading Response, *MSt*—Mid-Stance, *TSt*—Terminal Stance, *PSw*—Pre-Swing, *ISw*—Initial Swing, *MSw*—Mid-Swing, *TSw*—Terminal Swing.

To deliver stimulation at the desired times in the gait cycle, the stimulator trigger was modified to compensate for the inherent gait phase detection delays associated with the GPD system. Five stimulator triggers (*T1*–*T5*) were tested. *T1*: A pre-trigger strategy where the current detected gait phase triggered stimulation associated with the next gait phase. *T2*–*T4*: A pre-trigger strategy where the stimulation associated with the upcoming gait phase was triggered after a certain percentage of the current gait phase had passed. The time associated with percentage delay was gait phase dependent and equal to a percentage of the average duration of the gait phase (*T2*: 25% gait phase duration, *T3*: 50% gait phase duration, and *T4*: 75% gait phase duration) (Figure 1). Gait phase duration was calculated using a moving average and included ten previous gait cycles; initialization of the system required 10 gait cycles to be collected before stimulation delivery began. *T5*: Onset detection of current gait phase triggered stimulation associated with the current phase.

Stimulation delivery was simulated for the five triggers by recording stimulation signal in ten analog channels of a data acquisition board (NI-USB-6218, National Instrument, Austin, TX, USA) integrated into the motion capture system. The analog channels were representative of stimulation applied to the key lower extremity muscle groups (plantarflexors, dorsiflexors, quadriceps, hamstrings, and gluteals) on the left and right sides.

### 2.2. Experimental Protocol

Seven typically-developing (TD) children (5 Females, 12.4 ± 2.15 years old) were recruited locally and Temple University IRB-approved parental consent and child assent documents were obtained prior to subject participation (protocol # 20459). Prior to the gait analysis, each subject’s self-selected walking speed was determined over ground with the 10 Meter Walk Test (MWT) [49] and height and weight were collected. Subject’s self-selected walking speed was used to set the treadmill speed; however, if the subject identified that the self-selected speed felt incorrect on the treadmill, the treadmill speed was adjusted based on subject feedback. The subject donned the sensors while walking on a split-belt, instrumented treadmill (Bertec, Columbus, OH, USA). After a treadmill-walking accommodation period [50], a 30 s walking trial was collected. Kinematic and kinetic data were captured using an 8-camera motion capture system (Motion Analysis Corporation, Santa Rosa, CA, USA) and two force plates (Bertec, Columbus, OH, USA), respectively.

**Figure 2:**
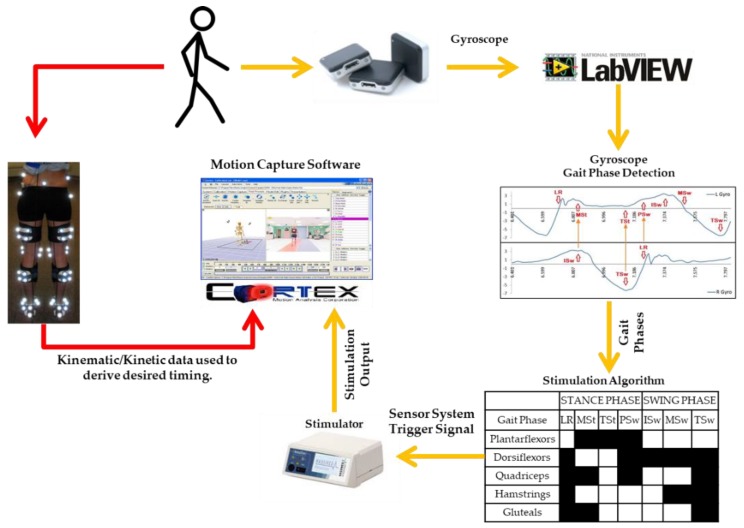
Schematic of the comparison between the stimulation signal of the FES system (yellow arrows) and the desired stimulation timing derived from motion capture data (red arrows).

The schematic in Figure 2 illustrates the output generated from the FES system and the reference timing determined from motion capture. While the participants walked on a treadmill at a comfortable speed, the FES system generated a stimulation signal (stimulation output). Rather than delivering stimulation to the subject, this signal, as previously described, was collected through analog channels that were integrated into the motion capture system, similar to the way EMG is recorded in the motion capture system. This provided stimulation signals that were synchronized with the kinematic and kinetic data and the subjects themselves did not receive FES. Kinematic, kinetic, and stimulation signal data were processed in Visual 3D (C-Motion, Germantown, MD, USA). Data were normalized to a gait cycle. Events [23,28] associated with the initiation of each gait phase were identified in kinematic and kinetic data and the *desired* timing of stimulation corresponding to the gait phases were indicated. An amplitude threshold method was used to convert the stimulation signal generated by the FES system from analog signals (volts) into binary signals. The on and off times of stimulation, normalized to a gait cycle (% GC), were determined for each muscle group and gait cycle. 

### 2.3. Statistics

The duration of each gait phase (*LR*, *MSt*, *TSt*, *PSw*, *ISw*, *MSw*, and *TSw*) was summarized using mean ± SD. The average stimulation signal onset time and duration, as a percent of the gait cycle (% GC), for the desired timing (*DESIRED*) and five trigger conditions (*T1*, *T2, T3*, *T4*, and *T5*) were calculated for each muscle group. Muscle groups included were gluteals (*G*), hamstrings (*H*), plantarflexors (*PF*), dorsiflexors (*DF*), and quadriceps (*Q*). Stimulation to the quadriceps was applied twice during a gait cycle; therefore, this muscle group was separated into two groups for analysis. *Q* represents stimulation to the quadriceps from *TSw* to *MSt* and *Q2* represents stimulation to the quadriceps from *PSw* to *ISw*. Difference in stimulation onset time was calculated between the desired timing (*DESIRED*) and each trigger condition (*T1*, *T2, T3*, *T4*, and *T5*). Trigger performance was summarized as the mean (±SD) and range of the difference in stimulation onset time across all muscle groups. 

The optimization approach leveraged signal detection performance measures [51] applied to the alignment of the stimulation signal with the desired stimulation time. In those terms, a true positive (TP) represents the duration, measured as a percentage of gait, of stimulation signal aligned with desired stimulation time; a false positive (FP) represents the duration of stimulation not aligned with desired stimulation time; a false negative (FN) represents the duration of desired stimulation time not aligned with stimulation signal; and a true negative (TN) represents the remaining percentage of the gait phase. These four can be further summarized as *recall*, TP/(TP + FN); *precision*, TP/(TP + FP); and their harmonic mean *F1*, 2 × *recall* × *precision/*(*recall* + *precision*). These summary measures, with an emphasis on the combined measure *F1*, served as responses in an analysis to determine which of the five trigger conditions was optimum, in maximizing *recall*, *precision*, and *F1*.

A standard analysis of variance (ANOVA) was performed on the response *F1* using JMP^®^Pro 14.0.0. The *F1* measure was chosen because of its role in combining precision and recall in one measure. It is easily interpretable, ranging between 0 (worst) and 1 (best). An ANOVA approach was employed to partition the variability attributable to model terms formed over a full factorial crossing of the random factor, participant (7 levels), and fixed factors of muscle group (6 levels), side (2 levels), trigger condition (5 levels), and their interactions. Ten repetitions of each condition were planned, 4200 observations in all. Of these, 135 were lost, but no treatment condition saw fewer than 7 observations. Restricted maximum likelihood (REML) estimation was used to account for the slight data imbalance involving a design with a random factor. ANOVA post hoc analysis (Tukey HSD), with variability appropriately partitioned, was leveraged to explore optimality among the candidate trigger conditions: *T1*, *T2*, *T3*, *T4*, and *T5*. Differences observed among model-derived least squares means profiled over start time provided a basis for start time preference.

## 3. Results

Delivery of stimulation was evaluated for five trigger conditions (*T1*, *T2*, *T3*, *T4*, *T5*). Triggers were initiated by onset of gait phase detection and subject specific gait phase delay durations. Subject specific gait phase delay durations were calculated as an operator-defined percentage of the subject’s average gait phase duration of 10 previous gait cycles. The percentages that corresponded with *T1* and *T5* were 0% and 100%, respectively. Average (±SD) phase durations varied between gait phases (Table 2), illustrating that triggers had a different delay time associated with each gait phase.

Twenty-seven out of 840 sample sets were excluded from the stimulation timing averages because of missing start/stop times for at least one trigger conditions. Almost half of the exclusions (13 records) were associated with stimulation to the quadriceps during *PSw* (*Q2*). Six *DF* samples were excluded and the remaining eight were equally distributed between *G*, *H*, *PF*, and *Q* muscle groups.

**Figure 3:**
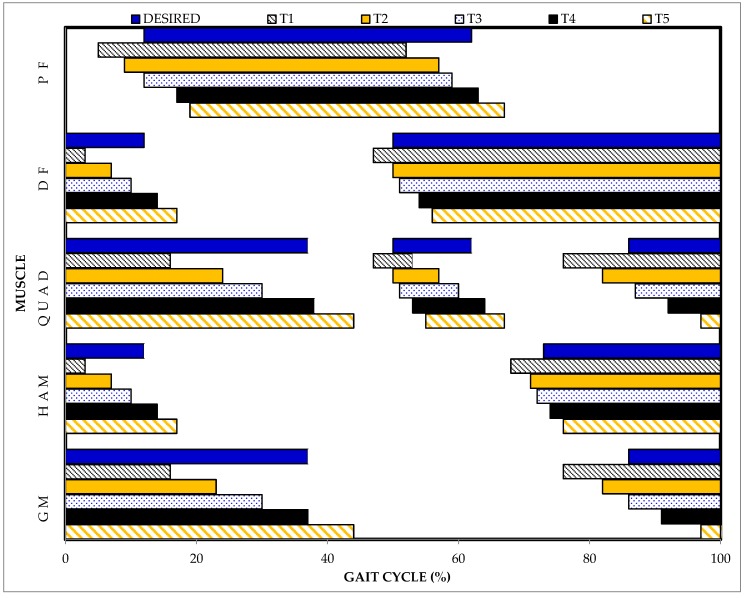
Comparison of desired stimulation timing determined from motion capture data (DESIRED) and the stimulation timing for five finite-state triggers used to control a FES system during walking. *T1*: Current gait phase-triggered stimulation for upcoming phase (pre-trigger), *T2*: 25% gait phase duration delay added to pre-trigger, *T3*: 50% gait phase duration delay added to the pre-trigger, *T4*: 75% gait phase duration delay added to the pre-trigger, and *T5*: Current gait phase-triggered stimulation for current phase.

Figure 3 illustrates the average stimulation timing of the five trigger conditions compared to the desired stimulation timing (*DESIRED*) for each muscle group and Table 3 provides descriptive statistics of the trigger conditions. *T1* and *T5* produced stimulation signals that started 6.5 ± 4.4% GC earlier and 7.0 ± 5.2% GC later than the desired stimulation timing, respectively. The difference between the stimulation signal and desired stimulation onset timing had ranges of 36% and 40% GC for *T1* and *T5* triggers, respectively.

The differences in stimulation delivery onset time compared to the desired time were proportional to the percent delays added to the gait phase detection onset. *T3* produced stimulation signals that started closest to the desired stimulation timing. The average onset time difference between the stimulation signals and desired stimulation was 0.3 ± 4.1% GC and had a range of 28% GC; stimulation signals occurred 13% GC earlier and 14% GC later than the desired stimulation time. The onset timing difference was −2.3 ± 4.3% GC when *T2* was used to trigger the stimulator and indicated that, on average, the stimulation signals occurred prior to the desired stimulation timing. The onset of the stimulation signals ranged from occurring 13% GC earlier to 11% GC later than the desired stimulation time. *T4* produced stimulation signals with an average onset time difference of 3.9 ± 4.6% GC and a range of 55% GC.

When detection of *TSw* occurred very close to the end of a gait cycle, *T4* and *T5* were associated with stimulation signals that started in the next gait cycle (Table 3). When *T4* triggered stimulation to *G* and *Q,* six of 276 stimulation onsets were delayed into the next gait cycle. Sixty-six out of 276 stimulation onset times were delayed into the next gait cycle with *T5*. When *T1* triggered the termination of stimulation to *H* and *DF*, 11 and 13 stimulation stop times ended in the *TSw* phase of the previous gait cycle (Table 3).

A summary of trigger performance is seen in Table 4. Generally, *T3* and *T4* show the best values for each summary performance measure, with little difference between them. With emphasis given the combined measure *F1*, the preference might be given to *T4*, but there is no statistically significant difference between them.

The analysis of *F1* incorporating all the data found significant differences in main effects among start times (*p* = 0.0003), muscle groups (*p* < 0.0001), and their interaction (*p* < 0.0001). Post hoc comparisons (α = 0.05) for trigger condition found differences only between *T1* and all others. Post hoc comparisons of means for muscle groups found no difference in *F1* means for *DF* and *PF*, *PF* and *H*, and *G* and *Q*. All other muscle group comparisons were statistically significant (α = 0.05). The order of *F1* means was *DF* (0.919), *PF* (0.883), *H* (0.873), *G* (0.816), *Q* (0.816), and *Q2* (0.548). The most striking aspect of interaction related to optimality was that stimulation timing to *DF* and *PF* was relatively stable over all trigger conditions; whereas, stimulation timing to other muscle groups were more pronounced in their adverse response to trigger condition other than *T3* or *T4*.

## 4. Discussion

A multichannel functional electrical stimulation (FES) system, with flexible finite-state control and capability to stimulate 10 lower extremity muscle groups during a gait cycle, was successfully developed by integrating two wireless sensors, two six-channel stimulators, and custom software. Most systems in the literature had low gait phase detection resolution regardless of the sensors used (i.e., FSRs [16,17,20,22,53,54,55], tilt sensors [56,57], or inertial sensors [42,45]); this limited the amount of control over stimulation delivery during a gait cycle. The reduced number of finite states also influenced the timing accuracy of stimulation to certain muscle groups, such as the gluteals and quadriceps. The GDP system [47] utilized in this study provides flexible trigger control, driven by detection of all seven gait phases, and allows for more appropriately-timed stimulation delivery to as many as five muscle groups, bilaterally, governed only by the number of channels available on the stimulator.

Although the gait phase detection resolution of the GPD system [47] provides a trigger for every gait phase, it was inferred that inherent delays associated with the wearable sensor system would cause stimulation timing errors. Sources of timing delay include latencies associated with wireless streaming of IMU data (~10–75 ms) [58], system timing indeterminacies associated with USB communication protocol (as high as 55 ms) [59], and Windows operating system not operating at a real-time capacity. Five FES trigger conditions were evaluated to determine compensations necessary to deliver stimulation that best matched the desired stimulation timing. In typically-developing children, *T3* and *T4* were identified as the most accurate finite-state controllers to trigger FES and produce appropriately-timed stimulation signals to the gluteal, hamstring, quadriceps, plantarflexor, and dorsiflexor muscle groups during a gait cycle. Both triggers applied a percent of gait phase duration delay to the current gait phase (*T3*: 50% gait phase duration delay, *T4*: 75% gait phase duration delay) and triggered stimulation for the upcoming gait phase. While *T3* was associated with an average stimulation onset time closest to the desired stimulation timing, the harmonic mean of recall and precision (*F1*) indicated that preference might be given to *T4*. *F1* was not significantly different between *T3* and *T4*.

Twenty-seven out of 840 sample sets were excluded from the stimulation timing evaluation because of missing start/stop times for at least one of the trigger conditions. Capturing these missing time points illustrates the importance of recording stimulation signals to uncover FES system issues that may occur between gait phase detection and stimulation delivery. The practical consequence of missing start/stop times, as well as time differences between the stimulation delivery and desired timing, is that a patient would not receive stimulation at the appropriate times.

Both trigger conditions (*T3* and *T4*) associated with stimulation delivery closest to the desired timing in typically-developing children were piloted in one adolescent with cerebral palsy (CP) (16-year-old, male). On average, the participant with CP had longer gait cycles (~1.4 s) than the typically-developing participants (~1 s). Additionally, average (±SD) gait phase durations differed from the typically-developing participants (*LR*: 204.2 ± 46.4 ms, *MSt*: 273.0 ± 60.1 ms, *TSt*: 231.5 ± 89.6 ms, *PSw*: 204.1 ± 46.4 ms, *ISw*: 78.8 ± 62.1 ms, *MSw*: 212.9 ± 94.6 ms, *TSw*: 234.1 ± 87.3 ms). The different distribution of time spent in each gait phase influenced the time added to the percent delays for *T3* and *T4*. The average stimulation onset timing difference was −6.2% GC for *T3*. Stimulation occurred prior to the desired timing. The difference ranged from occurring 10% to 2% GC earlier depending on the targeted muscle group. The *T4* condition was associated with stimulation signals closest to the desired time. The average stimulation onset difference was 1.5% GC and had a range of 7% GC; stimulation signals occurred 5% GC earlier and 2% GC later than the desired time. Although different than the average stimulation onset timing results observed in the typically-developing children, these results illustrate the feasibility of implementing FES trigger compensation strategies based on gait detection in a population with atypical gait.

One limitation of utilizing a finite-state controller is the input resolution is too low to provide the level of feedback necessary for real-time modulation of stimulation parameters to compensate for muscle fatigue. With the capability of off-line adjustments; however, the system allows for modifications that help to address muscle fatigue. For example, the use of variable frequency trains known to preserve force in FES applications [31] may be implemented; this feature is not currently available in commercial systems. Furthermore, stimulation pulse duration and current amplitude can be manually adjusted in real-time to account for declining gait function associated with fatigue. Even with declines in walking function, the system’s gait phase detection [47] is robust enough to detect distinct gait phases to use as the trigger for FES. There are potential mobility limitations with the use of a tethered FES system; there is a wired connection between the surface electrodes and stimulator. The participants are limited to treadmill walking or donning the cumbersome stimulators during over-ground ambulation. There is also the risk of tripping on the electrode cables and their length limits where stimulators can be placed.

An experimental limitation of this evaluation was assessment of stimulation timing solely during treadmill walking. Although trigger compensations were successfully applied based on gait phase detection when subjects walked at constant speeds on the treadmill, potential variations in stimulation delivery time is unknown in different environments such as over-ground walking. Further evaluation of the system’s performance during over-ground walking, or during perturbations to the limb while walking, may provide more insight into the robustness of the system’s trigger compensation strategy. Lastly, deploying multiple trigger compensation strategies, specific to gait phase and/or targeted muscle group, may further improve stimulation onset timing differences and system performance measures.

## 5. Conclusions

The stimulation delivery timing measures of the FES system contribute to the evidence that utilizing gait phase detection for finite-state control is a viable method for triggering stimulation and the accuracy of the stimulation timing is likely greater than open-loop systems. These measures also illustrate the importance of validating the stimulation delivery time associated with the stimulation trigger of the FES system to verify that appropriate compensations are made for system delays.

## Figures and Tables

**Table 1:** Stimulation program based on typical muscle firing patterns during gait [30]. Black boxes illustrate when the muscle groups were stimulated during the gait cycle. *LR*—Loading Response, *MSt*—Mid-Stance, *TSt*—Terminal Stance, *PSw*—Pre-Swing, *ISw*—Initial Swing, *MSw*—Mid-Swing, *TSw*—Terminal Swing.

	Stance Period				Swing Period		
Gait Phase	*LR*	*MSt*	*TSt*	*PSw*	*ISw*	*MSw*	*TSw*
Plantarflexors							
Dorsiflexors							
Quadriceps							
Hamstrings							
Gluteals							

**Table 2:** Average (±SD) gait phase duration during walking in typically-developing children. Subject specific phase duration was used to calculate the trigger percent delay for each gait phase.

Gait Phase	Average (±SD) Duration (ms)	Average (±SD) Duration (% GC)	Rancho Los Amigos Duration (% GC) [52]
*LR*	133.8 ± 21.8	12.2 ± 2.0	12
*MSt*	266.8 ± 21.4	24.3 ± 2.8	19
*TSt*	144.3 ± 18.4	13.2 ± 1.5	19
*PSw*	133.9 ± 21.6	12.0 ± 2.0	12
*ISw*	120.0 ± 17.9	11.0 ± 1.9	13
*MSw*	139.2 ± 13.4	12.7 ± 1.4	12
*TSw*	150.0 ± 18.4	13.9 ± 1.4	13

**Table 3:** Descriptive statistics of desired stimulation timing (DESIRED) and the stimulation timing for five trigger conditions as a percentage of the gait cycle (% GC).

Muscle Group		Start Time (% GC)						Stop Time (% GC)					
		Desired	T1	T2	T3	T4	T5	Desired	T1	T2	T3	T4	T5
G	Avg	86	76	82	86	91	97	37	16	23	30	37	44
	SD	1	3	4	4	4	4	2	4	5	4	5	4
	Max	91	85	92	97	100	9*	43	25	39	40	53	56
	Min	81	67	71	75	81	86	33	4	13	18	25	31
H	Avg	73	68	71	72	74	76	12	3	7	10	14	17
	SD	2	4	5	5	4	5	2	3	4	4	7	4
	Max	77	81	86	91	90	90	17	13	17	26	53	29
	Min	68	56	59	59	63	63	9	96 **	0	2	5	9
Q	Avg	86	76	82	87	92	97	37	16	24	30	38	44
	SD	1	4	4	4	3	4	2	4	5	4	6	5
	Max	91	85	92	97	100	9 *	43	25	40	41	66	66
	Min	81	57	72	76	82	86	33	1	13	19	26	32
Q2	Avg	50	47	50	51	53	55	62	53	57	60	64	67
	SD	2	4	4	4	4	4	2	3	4	4	4	5
	Max	55	58	63	64	64	69	68	64	70	73	74	79
	Min	44	38	41	41	43	44	56	47	49	51	57	57
DF	Avg	50	47	50	51	54	56	12	3	7	10	14	17
	SD	2	4	4	4	4	5	2	3	4	4	4	4
	Max	55	58	64	64	76	74	17	16	17	27	24	29
	Min	44	38	41	41	43	44	9	96 **	0	2	6	9
PF	Avg	12	5	9	12	17	19	62	52	57	59	63	67
	SD	2	3	4	4	7	4	2	3	4	4	4	5
	Max	17	13	18	28	55	31	68	64	69	73	73	100
	Min	9	0	2	5	7	10	56	47	49	51	57	57

* Indicates a stimulation signal that started in the next gait cycle. ** Indicates a stimulation signal that ended in *TSw* of the previous gait cycle.

**Table 4:** Performance measures over all observations by trigger condition.

		Trigger Condition				
		T1	T2	T3	T4	T5
Recall	Mean	0.635	0.753	0.820	0.846	0.795
	SD Error	0.008	0.007	0.006	0.005	0.006
Precision	Mean	0.767	0.888	0.928	0.916	0.830
	SD Error	0.008	0.005	0.004	0.005	0.006
F1	Mean	0.716	0.808	0.866	0.878	0.819
	SD Error	0.006	0.006	0.005	0.005	0.005
